# Physical Impact of Traditional and Virtual Physical Exercise Programs on Health Outcomes among Corporate Employees

**DOI:** 10.3390/jcm13030694

**Published:** 2024-01-25

**Authors:** John Oginni, Grace Otinwa, Zan Gao

**Affiliations:** 1 Department of Kinesiology, Recreation, and Sport Studies, The University of Tennessee, Knoxville, TN 37996, USA; joginni@vols.utk.edu; 2 Department of Human Kinetics and Health Education, University of Lagos, Lagos 101017, Nigeria; gotinwa@unilag.edu.ng

**Keywords:** blood pressure, body composition, heart rate, remote exercise, web-based exercise, waist circumference

## Abstract

**Background**: Technology-based exercise programs have the potential to reduce chronic disease prevalence and obesity-related risks. This research focuses on the impact of both traditional and virtual exercise programs on the health metrics of corporate employees. **Methods**: The study involved 30 corporate employees (16 females, average age ± SD = 37.8 ± 8.8 years) from Nigeria, who were divided into two experimental groups (one experiencing traditional exercises with an on-site trainer, and the other participating in live, virtual classes via Zoom) and a control group continuing usual practices. The 6-week intervention measured several health outcomes, including resting heart rate, blood pressure (both diastolic and systolic), body mass index (BMI), waist circumference, and waist-hip ratio, both before and after the intervention. **Results**: One-way analysis of covariance yielded significantly greater decreases in the diastolic blood pressure, F(2,27) = 3.7, *p* < 0.04; systolic blood pressure F(2,27) = 3.5, *p* < 0.04, body mass index, F(2,26) = 7.8, *p* < 0.01, and waist circumference, F(2,27) = 39.1, *p* < 0.01. **Conclusions**: The study concludes that virtual-based exercise programs are as effective as traditional ones. Offering a virtual exercise option provides flexibility, potentially increasing adherence to exercise routines among corporate workers.

## 1. Introduction

The health system is one of the key sectors severely impacted by the COVID-19 pandemic [1,2]. An efficient health system aims to meet the health needs and expectations of the population, with a focus on improving individual health [3]. Chronic diseases are a significant concern, especially in low- and middle-income countries, accounting for over three-quarters of global chronic disease deaths [4,5]. A major global public health issue is insufficient physical activity [6,7,8], which affects more than 80% of adolescents, and approximately a quarter of adults are deemed insufficiently active. These statistics have exhibited little change over the past two decades [9]. Busy lifestyles and heavy workloads contribute to physical inactivity [8]. Common barriers to engaging in physical activity include time constraints, lack of access to facilities or equipment, and a limited knowledge of safe, effective physical activity methods [10,11]. Physical activity, defined as any movement increasing energy expenditure above resting levels, and exercise, a planned physical activity, are known to improve health and fitness across various age groups and health conditions [10,12]. Contemporary interventions leveraging technology employ methods such as web-based platforms, text messaging, smartphone applications, and social media-fused podcasts [13,14]. These approaches capitalize on the ubiquity of technology to encourage healthier behaviors, showcasing the capacity of digital tools in fostering active lifestyles [15]. Mobile health (mHealth), as defined by the World Health Organization, involves medical and public health practices supported by mobile devices [4,16]. mHealth coaching is gaining traction, offering advantages over traditional methods in terms of accessibility, scalability, cost-efficiency, and adaptability [17]. The evolution of technology, including wearable devices, apps, and advanced data analytics, has markedly altered the mHealth intervention landscape [18]. This development has not only facilitated but also enhanced the efficacy of health promotion interventions. The growing acceptance of these interventions is largely fueled by increased access to and ownership of tech gadgets, particularly smartphones [19]. Studies have shown that mHealth tools like apps and wearables are effective in promoting physical activity and achieving favorable results, such as weight loss, lowered BMI, better dietary compliance, and reduced sedentary habits, especially when combined with other intervention elements [19,20].

White-collar professionals, particularly corporate workers, often engage in light-intensity physical activity and sedentary behaviors, leading to increased BMI and blood pressure [8,21,22]. Previous studies have identified that employees are at a higher risk of chronic diseases due to sedentary lifestyles [23,24]. However, physical exercise programs implemented at the workplace have demonstrated numerous benefits, such as improved flexibility, reduced musculoskeletal disorders, and overall enhancement in quality of life [25]. In line with this, research by Moreira et al. (2022) indicated that online workplace exercise programs also have positive impacts, leading to better physical function, enhanced performance, and improved quality of life. Additionally, online lifestyle interventions have proven effective in reducing psychological stress, which is associated with improvements in various health outcomes for employees [26]. Interventions that leverage digital platforms for physical exercise have proven to be suitable for meeting physical activity (PA) recommendations within specific populations [27]. However, a systematic review indicates limited evidence regarding the effectiveness of virtual workplace interventions in terms of health-related outcomes [28].

Smartphone ownership is on the rise in Sub-Saharan Africa (SSA), notably in South Africa, Kenya, Nigeria, Senegal, and Ghana [29]. Despite this trend, less than half of SSA countries employ mHealth platforms, and initiatives are limited across various health areas [30]. The lack of extensive scientific research on the viability of mHealth interventions for encouraging physical activity and exercise among adults and corporate employees in Sub-Saharan Africa (SSA), especially in Nigeria, is apparent. Consequently, this study aims to fill the existing gap by addressing the lack of information on mHealth interventions designed to promote physical activity and exercise participation among the adult population of SSA, with a specific focus on Nigeria. Top of FormIn 2024, worksite health promotion claims the second spot among the top twenty fitness trends [31]. This revealed a strong interest in elevating overall health and quality of life in corporate employees, likewise in workplace productivity. Therefore, the purpose of this study is to investigate the impact of both traditional and virtual physical exercise programs on the health outcomes of corporate workers, exploring the effectiveness of these interventions in a region with scarce research. This study aims to contribute insights into the use of technology-driven methods for enhancing physical exercise and overall health outcomes.

## 2. Materials and Methods

### 2.1. Participants

In this research, 30 corporate workers (16 females, average age 37.8 ± 8.8 years) voluntarily participated, forming a convenience sample. Recruitment was performed informally through word-of-mouth, this included speaking to small gatherings about the research study within an educational institution. Inclusion criteria for eligibility were: being a corporate worker of an institution; willingness to provide consent and accept randomization; basic English communication capabilities; possession of an Android or IOS operating system smartphone with a reliable internet connection. Participants were excluded if they were pregnant, at risk of injury or ill health from their increasing physical activity (as assessed by the Physical Activity Readiness Questionnaire), or if they already met the physical activity recommendations (as assessed via a single item asking if participants participated in 30 min of physical activity on most days of the week). The study’s data collection phase occurred between August and October 2021. Participants were randomly divided into three groups, with ten individuals each in the physical exercise group (onsite), the virtual exercise group, and the control group. It is noteworthy that all participants were educators affiliated with The Bells Schools in Ogun State, Nigeria.

### 2.2. Procedures

Prior to their participation, all participants underwent the informed consent process and completed the Physical Activity Readiness Questionnaire [32]. The study received ethical approval from the Department of Human Kinetics and Health Education’s postgraduate ethics committee at the University of Lagos on 6 September 2021. An introductory letter was also obtained from the department to aid in engaging and collaborating with the target population for data collection. The study’s procedures conformed to the ethical standards of the institution and/or national research committee, aligning with the Declaration of Helsinki and its subsequent modifications [33].

### 2.3. Measures

The selected measurements were influenced by significant findings in the literature regarding common health indicators that impact corporate workers with limited physical activity. Furthermore, measurements were taken before and after the intervention, with participants in light attire. Participants in the virtual group were instructed to download and set up the Zoom software (version 5.7.5), receiving technical support as needed. Data collection was conducted by a skilled research assistant using specific testing equipment.

### 2.4. Blood Pressure and Heart Rate

Blood pressure and heart rate were measured using an Andon digital automatic blood pressure monitor (Andon KD 595, Andon Health, Tianjin, China), validated per the European Society of Hypertension International protocol. Participants were instructed not to talk and seated quietly for at least 5 min in a chair with back support with their feet on the floor and their arms supported at heart level. The appropriate cuff size was firmly wrapped around the upper arm at heart level, with alignment with the brachial artery. Two measurements were taken with a rest of 1 min between the first and second measurements [34,35]. The resting diastolic, systolic, and heart rate were recorded in the datasheet.

### 2.5. Waist and Hip Circumferences

These were assessed using a flexible and inelastic 1 × 120 tape measure. For the waist circumference, the participant assumes a standing position with arms positioned alongside, feet in close proximity, and a relaxed abdomen. A horizontal measurement is then obtained at the narrowest section of the torso, specifically located above the umbilicus and below the xiphoid process [34,35]. and recorded in inches. Likewise, for the hip circumference, the participant assumes a standing position with arms positioned alongside, feet in close proximity, and the measurement taken at the maximal circumference of the buttocks. The waist-hip ratio for all participants was calculated by dividing the waist circumference by the hip circumference.

### 2.6. Body Mass Index

Participants’ weight was determined using a Hana weighing scale and a fixed stadiometer for height measurement. Prior to both weight and height measurements, participants were instructed to remove shoes, socks and heavy clothing. For measuring weight, participants were instructed to stand on the middle of the scale, and their weight was noted down to the nearest decimal point. To measure height, participants were asked to stand with their feet together, flat against the wall. The research assistant ensured that the participants’ legs were straight, arms rested at their sides, and shoulders were kept level during this process. Body Mass Index (BMI) was then calculated based on the square of their height and weight.

### 2.7. Physical Activity Readiness Questionnaire (PAR-Q+)

In this study, we utilized the PAR-Q+ (2021), a seven-step questionnaire accompanied by additional follow-up sections. This questionnaire is designed to assess the presence of risk factors related to moderate physical activity and evaluate the severity of certain medical conditions.

### 2.8. Intervention Conditions

Participants in the traditional group underwent a physical exercise intervention program led by a trained exercise leader, whereas the virtual group also engaged with a trained exercise leader via the Zoom application. The trained exercise leader was a graduate student of exercise physiology with experience in group exercise training to ensure proficiency in administering the exercise sessions. The exercise sessions were conducted over a 6-week period. Conversely, the control group did not receive any intervention program and were asked to continue their normal lifestyle behaviors. Program attendance was monitored virtually by the exercise leader through the Zoom App for the virtual group, and on-site for the traditional group throughout the program. The physical exercise program, adhered to the guidelines of the American College of Sports Medicine [34] for aerobic exercise prescription with some modifications. Each session was conducted two days per week, lasted for 60 min over the course of 6 weeks for both intervention groups. The physical activity program commenced with a 5–10 min warm-up, followed by 30–40 min for conditioning aerobic exercises. Subsequently, a cool-down, and stretching exercises which spanned between 7–10 min concluded each session. Aerobic exercises included jogging (jogging in place for the virtual group), aerobic dance, aerobic steps, regular or modified jumping jacks, and other exercises used in group conditioning classes. Exercise sessions started at a light intensity, with the exercise leader periodically engaging participants (e.g., every 3–5 min) to assess exercise intensity using the Talk Test [10,36] and to keep participants at a level below exhaustion. Aerobic exercise progression initially included increasing the duration by no more than 10% per week and followed by an increase in intensity as appropriate [34,36,37]. The exercise leader placed emphasis on demonstrating exercises to participants and then giving feedback to ensure proper form on the exercises. Participants could therefore develop competence that they were doing exercises correctly and effectively. Notably, both intervention groups followed a comparable exercise program.

### 2.9. Statistical Analysis

Data were analyzed using IBM-SPSS 22.0. (IMN Inc.; Armonk, NY, USA). Descriptive statistics were calculated for age and gender. Thereafter, analysis of covariance (ANCOVA) and baseline BMI as the covariate evaluated differences between groups and the selected outcomes. Finally, the post hoc Bonferroni analyses were performed to further examine differences between the three groups for the health outcomes. The study level of significance was set at 0.05.

## 3. Results

Outcomes assessed include, blood pressure, resting heart rate, waist circumference, waist–hip ratio, and BMI following a standardized procedure as highlighted in the method section. The analysis of covariance (ANCOVA) revealed significant differences in several outcomes. Specifically, there were notable effects on systolic blood pressure (Sys), F(2,27) = 3.5, *p* < 0.04, and diastolic blood pressure (Dia), F(2,27) = 3.7, *p* < 0.04. Additionally, waist circumference (WC) showed a remarkable change, F(2,27) = 39.1, *p* < 0.01, and body mass index (BMI) exhibited significant alterations, F(2,26) = 7.8, *p* < 0.01 (Table 1). It is noteworthy that preliminary analysis shows no significant differences on those variables between the intervention group and control group (Sys *p* = 0.99, Dia *p* = 0.77, WC *p* = 0.68, BMI *p* = 0.19).

However, no significant effects were observed in resting heart rate (F(2,27) = 2.47, *p* = 0.10 and waist-hip ratio (F(2,27) = 0.50, *p* = 0.61, when compared to the control group.

Detailed Bonferroni analyses, conducted with a 95% confidence interval, indicated significant reductions in diastolic blood pressure. In the physical exercise group, there was a decrease ranging from 17.8 to 0.6 mmHg (*p* < 0.032) compared to the control group. Similarly, the virtual group showed a reduction ranging from 17.1 to 1.3 mmHg (*p* < 0.045) when compared to the control group. For systolic blood pressure, the physical group exhibited a decrease ranging from 15.1 to 1.6 mmHg (*p* < 0.035), and the virtual group showed a reduction ranging from 16.6 to 0.5 mmHg (*p* < 0.042), both in comparison to the control group. Regarding BMI, the physical activity group demonstrated a reduction of 2.9 to 0.6 kg/m^2^ (*p* < 0.02) relative to the control group, and the virtual exercise group exhibited a decrease of 2.6 to 0.3 kg/m^2^ (*p* < 0.009) compared to the control group. Furthermore, waist circumference in the physical group was 5.7 to 3.0 inches lower (*p* < 0.001) than the control group, and in the virtual group, it was 4.6 to 1.9 inches lower (*p* < 0.001) compared to the control group.

## 4. Discussion

This study aimed to assess the impact of traditional and virtual physical exercise programs on the health of corporate employees, addressing the existing knowledge gap regarding mHealth interventions for promoting physical activity and exercise in a region with limited research. The results were noteworthy, revealing significant improvements in diastolic and systolic blood pressure, waist circumference, and BMI among participants in both traditional and virtual exercise groups compared to the control group. These findings highlight the potential effectiveness of virtual exercise programs for the working population. However, no significant differences were observed in waist–hip ratio and resting heart rate between the intervention groups and the control group.

Interestingly, contrary to our initial expectations, neither the traditional nor virtual interventions significantly improved the resting heart rate over the six-week period. This outcome is in line with the findings of Gotink et al. [38], who also did not observe significant changes in heart rate following online mindfulness exercise training. However, a study by Daveri et al. [39] contrasts the outcome, who observed significant changes in resting heart rate in a remote online training. This suggests that the duration of the exercise program is a critical factor, and extending the intervention period beyond six weeks might yield different results.

Moreover, a key observation was the notable reduction in both systolic and diastolic blood pressure among participants in both exercise programs. Specifically, Bonferroni analysis with a 95% confidence interval highlighted a reduction of 17.1–1.3 mmHg in diastolic blood pressure and 16.6–0.5 mmHg in systolic blood pressure in the virtual group compared to controls. This finding is consistent with a remote exercise training program [40] though in the children population who observed a significant reduction in all blood pressure parameters after the intervention. Prior research by Gotink et al. [38] and Lisón et al. [41], also supports the efficacy of web-based exercise interventions in lowering blood pressure. Furthermore, significant reductions in waist circumference and BMI were noted in the intervention groups. The virtual group, in particular, showed a decrease in BMI of 2.6–0.3 kg/m^2^ and in waist circumference by 4.6–1.9 inches when compared to the control group. These findings echo those of meta-analyses [42] and specific interventions [43,44] that have demonstrated similar reductions through internet-based exercise programs. However, it is important to note that there was no significant effect on the waist-hip ratio. This suggests that the length of the intervention matters, with longer interventions, potentially exceeding 12 weeks, likely being more effective in altering body composition, particularly the waist-hip ratio [10]. Furthermore, the absence of nutritional guidance in our study may have influenced the limited or negligible changes observed in body composition. In 2024, worksite health promotion ranked second among the top 20 fitness trends, indicating a strong emphasis on improving the overall health and quality of life for corporate employees, as well as enhancing workplace productivity. In this context, the current study holds significant importance as it contributes to the scientific understanding of health-promoting behaviors. These behaviors offer a range of benefits, including reduced insurance costs, increased workplace efficiency, and improved mental well-being. As worksite health promotion continues to gain recognition, this research provides valuable insights, highlighting its broader implications for both individual and organizational welfare.

The findings of this study warrant consideration within the framework of its limitations. Conducted amidst the backdrop of the COVID-19 pandemic, the research faced constraints imposed by mobility restrictions, limitations on gatherings, and the prevailing economic crises. These challenges significantly impacted various aspects of the research processes, notably the recruitment of participants and the determination of an optimal sample size. The use of a small sample size may affect the study’s internal and external validity, as highlighted by Faber and Fonseca [45]. This was due to inevitable constraints experienced during the COVID-19 pandemic. These constraints include government restriction policy on gathering and lack of access to a reliable internet connection. The primary goal of this study is to establish baseline data for the effectiveness of virtual-based physical exercise programs in sub-Saharan countries, such as Nigeria. To improve the external validity and enable broader generalization, future research in sub-Saharan regions should incorporate larger sample sizes. Moreover, the duration of this study, which lasted only 6 weeks, may influence its outcomes. This timeframe corresponds to the initial conditioning phase in training principles. For more comprehensive results, it is recommended that future studies extend the duration of physical exercise interventions to between three and eight months, aligning with the improvement stage and adhering to effective training requirements.

- The results of this study carry practical implications with real-world relevance. Firstly, they indicate that a virtual physical exercise program can be just as effective as an onsite physical exercise program in achieving positive outcomes related to blood pressure, waist circumference, and BMI. Secondly, the virtual exercise program offers a level of flexibility that may enhance adherence to exercise routines, particularly among corporate workers who often face time constraints. This flexibility can contribute to the adoption of health-promoting behaviors, ultimately leading to multifaceted benefits such as reduced insurance costs and increased workplace productivity. These findings highlight the potential of virtual exercise programs as a valuable tool for improving the health and well-being of corporate employees.

## 5. Conclusions

In conclusion, this study sheds light on the impact of both traditional and virtual-based physical exercise programs on health-related outcomes. A significant takeaway is that virtual physical exercise programs can be as effective as onsite programs in achieving positive outcomes related to blood pressure, waist circumference, and BMI, offering a flexible option for the working population who juggle busy lifestyles and heavy workloads. Professionals and practitioners may opt for the implementation of remote physical exercise/activity (PA) programs, as these mobile health (m-health) interventions have demonstrated feasibility and cost-effectiveness in promoting health [18]. It is noteworthy that the current employment landscape is characterized by a blend of onsite and remote work hours. Consequently, the flexibility and accessibility offered by virtual physical exercise allows employees to leverage active physical behaviors to their advantage. This further strengthens the relevance of this study. Future research should consider longer intervention periods and include comprehensive psychological and social support measures to fully assess and enhance the benefits of virtual or remote physical exercise/activity programs [18,37,46]. It is also recommended for future studies to enhance sampling accuracy by diversifying participants across various segments of the working population.

## Figures and Tables

**Table 1:** Pre and post intervention of traditional and virtual physical activity program on health outcomes.

Outcome	Group	*n*	Test	Mean (S.D)	F	Adjusted R^2^	*p*
**Systolic Blood Pressure (mmhg)**	Physical	10 10	Pre-test Post-test	122.5 (18.96) 113.3 (16.1)	(2,22) = 3.5	0.15	0.04
	Virtual	10 10	Pre-test Post-test	127.50 (17.2) 119.0 (15.2)			
	Control	10 10	Pre-test Post-test	122.4 (19.4) 121.8 (21.2)			
**Diastolic Blood Pressure (mmhg)**	Physical	10 10	Pre-test Post-test	76.3 (7.9) 71.6 (7.2)	(2,27) = 3.7	0.16	0.037
	Virtual	10 10	Pre-test Post-test	79.40 (15) 73.10 (10)			
	Control	10 10	Pre-test Post-test	78.10 (6.2) 80.10 (5.6)			
**Waist Circumference (inches)**	Physical	10 10	Pre-test Post-test	34.2 (3.74) 31.6 (2.99)	(2,27) = 39.1	0.72	0.01
	Virtual	10 10	Pre-test Post-test	36.83 (7.94) 35.3 (7.9)			
	Control	10 10	Pre-test Post-test	35.25 (4.57) 37.05 (5.2)			
**BMI (kg/m^2^)**	Physical	10 10	Pre-test Post-test	27.7 (5.7) 26.2 (6.0)	(2,26) = 7.8	0.35	0.03
	Virtual	10 10	Pre-test Post-test	26.2 (5.5) 24.96 (4.8)			
	Control	10	Pre-test Post-test	24.85 (2.9) 25.09 (3.2)			

## Data Availability

Data are contained within the article.
